# Virulence and antibiotic resistance profile of avian *Escherichia coli* strains isolated from colibacillosis lesions in central of Algeria

**DOI:** 10.14202/vetworld.2019.1840-1848

**Published:** 2019-11-25

**Authors:** Nacima Meguenni, Nathalie Chanteloup, Angelina Tourtereau, Chafika Ali Ahmed, Saliha Bounar-Kechih, Catherine Schouler

**Affiliations:** 1 Laboratory of Analytic Biochemistry and Biotechnology, Mouloud Mammeri University, Tizi Ouzou 15000, Algeria; 2 ISP, INRA, Université de Tours, UMR 1282, 37 380, Nouzilly, France; 3 Regional Veterinary Laboratory of Draa Ben Khedda, Tizi Ouzou 15000, Algeria

**Keywords:** antibiotic resistance, avian *Escherichia coli*, extended-spectrum beta-lactamase, virulence

## Abstract

**Background and Aim:**

Avian pathogenic *Escherichia coli* cause extensive mortality in poultry flocks, leading to extensive economic losses. To date, in Algeria, little information has been available on virulence potential and antibiotics resistance of avian *E. coli* isolates. Therefore, the aim of this study was the characterization of virulence genes and antibiotic resistance profile of Algerian *E. coli* strains isolated from diseased broilers.

**Materials and Methods:**

In this study, 43 avian *E. coli* strains isolated from chicken colibacillosis lesions at different years were analyzed to determine their contents in 10 virulence factors by polymerase chain reaction, antimicrobial susceptibility to 22 antibiotics belonging to six different chemical classes and genomic diversity by pulsed-field gel electrophoresis (PFGE).

**Results:**

Mainly *E. coli* isolates (58.1%) carried two at six virulence genes and the most frequent virulence gene association detected were *omp*T (protectin), *hly*F (hemolysin) with 55.8% (p<0.001), and *iro*N, *sit*A (iron acquisition/uptake systems), and *iss* (protectin) with 41.8% (p<0.001). Some strains were diagnosed as virulent according to their virulence gene profile. Indeed, 23.25% of the isolates harbored *iro*N, *omp*T, *hly*F, *iss*, and *sit*A combination, 14% *omp*T, *hly*F, and frz_orf4_ (sugar metabolism), and 11,6% *iro*N, *hly*F, *omp*T, *iss*, *iut*A (iron acquisition/uptake systems), and *frz*_orf4_. The chicken embryo lethality assay performed on five isolates confirmed the potential virulence of these strains. All isolates submitted to PFGE analysis yielded different genetic profiles, which revealed their diversity. Overall, 97.2% of the isolates were resistant to at least one antibiotic and 53.5% demonstrated multi-antimicrobial resistance to three different antimicrobial classes. The highest resistance levels were against nalidixic acid (83.4%), amoxicillin and ampicillin (83.3%), ticarcillin (80.5%), pipemidic acid (75%), and triméthoprim-sulfamethoxazole (66.6%). For beta-lactam class, the main phenotype observed belonged to broad-spectrum beta-lactamases. However, extended-spectrum beta-lactamase associated with three at six virulence factors was also detected in 13 isolates. Two of them were attested virulent as demonstrated in the embryo lethality test which constitutes a real public threat.

**Conclusion:**

It would be imperative in avian production to discourage misuse while maintaining constant vigilance guidelines and regulations, to limit and rationalize antimicrobial use.

## Introduction

Poultry farming is undeniably the branch of animal production that has recorded a remarkable development in Algeria in recent years [1]. The poultry sector, which is 90% dominated by the private sector, has in less than a decade a significant leap with a considerable animal wealth of 240 million broilers and turkeys. However, the poultry industry still retains a dual character (industrial and artisanal models) [1]. In Algeria, as well as many other countries, colibacillosis is one of the most common bacterial infections in poultry and one of the main causes of mortality in chickens and turkeys, leading to significant losses in industrial poultry farming and carcass condemnations at slaughterhouse. The subgroup avian pathogenic *Escherichia coli* (APEC) is the etiologic agent of colibacillosis in chickens. Indeed, *E. coli* is a versatile species encompassing both commensals of the digestive tracts of many vertebrates, including humans, and pathogenic strains causing various intra- and extra-intestinal infections as a cause of airsacculitis, polyserositis, septicemia, poor growth performance, and carcass condemnation in affected flocks [2,3]. The disease-inducing potential of these isolates has been explained by the occurrence of specific virulence factors. Indeed, many virulence factors have been associated with APEC strains, although their role in the pathogenesis is not well known [4].

In animal production, antimicrobials are widely used as a growth promoter and in the treatment of infectious diseases. The use of antimicrobials in poultry production industries for the promotion of growth largely contributes to the high resistance to antimicrobial agents in normal flora of poultry and pathogenic microorganism [5].

Due to its ubiquity, *E. coli* has become one of the bacterial species that are commonly resistant to antibiotics and can transmit antibiotic-resistance genes from other *Enterobacteriaceae* species in the environment [6,7]. To date, little information has been available on characteristics of avian *E. coli* isolates in Algeria, especially their virulence factors content and antimicrobial resistance. Therefore, the aim of this study was the characterization of virulence genes and antibiotic resistance profile of Algerian *E. coli* strains isolated from diseased broilers.

## Materials and Methods

### Ethical approval

The tests on embryonated eggs do not require authorization from the ethics committee.

### Isolation of E. coli strains

A total of 43 chicken visceral organs (liver, lungs, heart, and spleen) were collected randomly between 2006 and 2013 from diseased chicken broilers from different poultry farms located in various regions of Central Algeria (Provinces of Bouira, Bejaia, Tizi Ouzou, and Boumerdes).

All farms were all-in, all-out intensive systems either in cages or on litter floors. Samples of these organs were cultured in brain and heart infusion broth then subcultured on MacConkey agar plates and on desoxycholate agar. The plates were incubated under aerobic conditions at 37°C for 72 h. *E. coli* isolates identification was confirmed by detecting *uid*A gene by polymerase chain reaction (PCR) [8].

### Virulence gene detection

Template DNA was prepared using the boiling method [9]. PCR reactions were performed according to published protocols [10,11]. The genes that were searched for were as follows: Adhesins (*pap*C and *fel*A), hemolysin (*hly*F), protectins (*iss* and *omp*T), iron acquisition/uptake systems (*iro*N, *iut*A, and *sit*A), component of a T6SS (*aec*26), and sugar metabolism (frz_orf4_). The *E. coli* strains used as positive controls in PCR assays were as follows: BEN 2908 [12,13] for *iut*A, *omp*T, *iss*, *sit*A, *aec*26, and frz_orf4_, MT189 for *fel*A, and BEN 2905 (J96) for *pap*C.

### Antimicrobial sensitivity testing

This test was performed by disk diffusion method on Mueller-Hinton agar using 22 antibiotic disk belonging to different antimicrobial classes including amoxicillin (30 µg), ampicillin (10 µg), amoxicillin/clavulanic acid (30 µg/disc), ticarcillin (30 µg), imipenem (10 µg/disc), aztreonam (30 µg/disc), cefazolin (30 µg), cefoxitin (10 µg), cefotaxime (30 µg), ceftazidime (30 µg), cefixime (30 µg), cefpirome (30 µg), kanamycin (30 µg), gentamicin (10 µg/disc), tetracycline (30 µg), sulfamethoxazole (1.25/23.75 µg), colistin (25 µg), nalidixic acid (30 µg), pipemidic acid (20 µg), ciprofloxacin (5 µg), ofloxacin (5 µg), and pefloxacin (5 µg). The presence of extended-spectrum beta-lactamases (ESBL) was detected by double-disc synergy method. Interpretation of the results was done according to CA-SFM procedures [14]. *E. coli* CIP7624 was used as quality control.

### Pulsed-field gel electrophoresis (PFGE)

PFGE was performed using the restriction enzyme *Xba*I as described by Moulin-Schouleur *et al*. [15]. Agarose plugs were prepared from a bacterial culture grown in brain heart infusion broth to an optical density (OD) at 600 nm of 1.0. After incubation for 2 h at 37°C in a lysozyme solution (10 mM Tris-HCl, pH 9, 100 mM ethylenediaminetetraacetic acid (EDTA), 5 mg/ml lysozyme, 0.05% sarkosyl), they were then incubated overnight at 55°C (without shaking) in a lysis solution (10 mM Tris-HCl, pH9, 100 mM EDTA, 1 mg/ml proteinase K, 1% sodium dodecyl sulfate) and washed 3 times for 1 h each time in TE buffer (10 mM Tris-HCl, pH 8, 1 mM EDTA).

For digestion, plugs were equilibrated in incubation buffer (Takara) containing 10 units *Xba*I restriction enzyme (Takara Bio Europe). PFGE was conducted in a CHEF-DRIII apparatus (Bio-Rad). Gels (1% agarose) were run at 14°C for 24 h in TBE buffer (4 mM Tris, 4 mM borate, 1 mM EDTA, pH 8.3) at 6 V/cm. PFGE was conducted in a CHEF-DRIII apparatus (Bio-Rad). The gels (1% agarose) were run at 14°C for 24 h in TBE buffer (Tris, 4 mM; borate, 4 mM; EDTA, 1 mM; pH 8.3) at 6 V/cm. The pulse times were increased from 10 to 30 s. As size markers, *Xba*I restriction fragments of *Salmonella enterica* serovar Braenderup H9812 were used. Cluster analysis using dice similarity indices was done in BioNumerics 6.6 software (at 0.5% tolerance and 0.5% optimization) (Applied Maths, Ghent, Belgium) to determine similarities and differences and to find or characterize the relationships among isolates.

### Chicken embryo lethality test

The method followed for this assay was described by Nolan *et al*. [16] and adapted by Trotereau and Schouler [17]. Virulence of five *E. coli* isolates (three ESBL and two broad-spectrum β-lactamases) was tested by the inoculation of washed bacterial cultures into the allantoic cavity of 11-day-old specific-pathogen-free chicken embryos. About 1.5 ml of overnight cultures of isolates grown in lysogeny broth (LB) at 37°C with shaking (180 rpm) were briefly centrifuged and resuspended in 1.5 mL of sterile/apyrogenic Dulbecco’s phosphate-buffered saline (DPBS). After the measurement of the OD at 600 nm, the inoculum was adjusted at a concentration of 10^3^ cfu/mL. The inoculation dose was confirmed by retrospective plating of serial dilutions onto LB agar plates. For inoculation, 100 µL of the diluted culture was administered into the allantoic cavity of 20 embryos per isolate and 10 eggs with 100 µL of sterile/apyrogenic DPBS. Embryos were candled once daily for 6 days post-challenge to monitor mortality. The data of survival were presented as Kaplan–Meier curves and analyzed using the log-rank test.

### Statistical analysis

The data were analyzed using multiple correspondence analysis (MCA) and p*-*values were calculated using a Chi-square test to find any significant relationship. p<0.05 was considered statistically significant. The proportion comparison Rprop test was performed using the statistical program R software (R Foundation for Statistical Computing, Vienna, Austria).

## Results

Identification of 43 isolates as *E. coli* was confirmed by the presence of the *uid*A gene in all isolates. PCR analysis for virulence factors of the 43 *E. coli* strains according to both [10,11] diagnostics showed that 55.8% of the isolates harbored *hly*F and *omp*T genes; 41.8% *iro*N, *iss*, and *sit*A genes. To a lesser extent, *iut*A genes and frz_orf4_ fragment were detected in 13.9% of the isolates and *fel*A gene on only 2.3% of the strains tested. In contrast, no isolate contained *aec*26 gene (Table-1).

**Table-1:** Virulence genes detected in avian pathogenic *Escherichia coli* isolates.

Virulence factor	Frequency	Percentage	p-value
*iroN*	18/43	41.80	1.203×10^−8^***
*iut*A	6/43	13.90	4.482×10^−15^***
*omp*T	24/43	55.80	2.89×10^−6^***
*iss*	18/43	41.80	1.203×10^−8^***
*hly*F	24/43	55.80	2.89×10^−6^***
*pap*C	1/43	2.30	2.2×10^−16^***
*fel*A	1/43	2.30	2.2×10^−16^***
*frz_orf4_*	6/43	13.90	4.482×10^−15^***
*aec*26	0	0	0
*sit*A	18/43	41.80	1.203×10^−8^***
No factor	18/43	41.80	1.203×10^−8^***

*** Very highly significant value. *iut*A (aerobactin siderophore receptor gene), *hly*F (putative avian hemolysin), *iss* (episomal increased serum survival gene), *iro*N (salmochelin siderophore receptor gene), and *omp*T (episomal outer membrane protease gene), *frz_orf4_ (sugar metabolism)*, *aec*26 (component of a T6SS), *sit*A (iron transport gene)

Otherwise, the strains had highly variable content of virulence genes. 11.6% of the isolates associated simultaneously up to six virulence factors and the association of *iro*N, *omp*T, *hly*F, *iss*, and *sit*A factors dominated with 23.25% (p<0.001). The *om*pT, *hl*yF, and frz_orf4_ combination was present in 14% of the strains, followed by *pap*C, *fel*A, *iro*N, *omp*T, *hly*F, and *iss* alone and associated with *iut*A in 2.3% of the strains studied. About 41.9% did not show any of the searched virulence factors.

### APEC diagnosis

**Figure-1:**
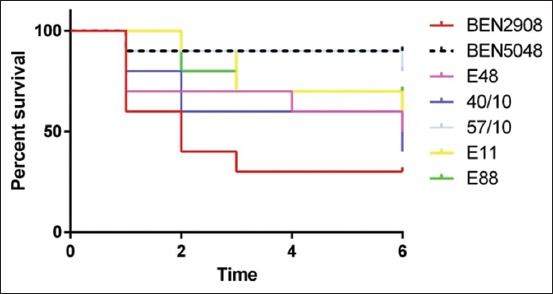
Chicken embryo test result on avian pathogenic *Escherichia coli* isolates. BEN 2908 APEC virulent strain; BEN 5048 negative control strains; E11, E48, and E88: ESBL profile; strains 40/10 and 57/10: broad-spectrum beta-lactamase profile.

*E. coli* strains (37.2%) were considered as potentially highly virulent APEC, according to Johnson *et al*. [10], which classify an *E. coli* as pathogenic based on the presence of minimum four of five virulence genes carried by plasmids associated with highly pathogenic APEC. Insight, the embryos mortality rate exceeding 40% after 3 days, the lethality test on chicken embryo confirmed the pathogenicity of the most tested isolates comprising two (E48 and E88) ESBL profile (Figure-1).

In contrast, all isolate strains were considered non-typable according to Schouler *et al*. [11]. Eighteen isolates were considered as non-pathogenic strains regarding these both diagnostics. The most prevalent pattern with 23.25% of strains associate five virulence genes *iro*N, *omp*T, *hly*F, *iss*, and *sit*A (Table-2). Regarding the search for virulence factors, high significant difference was found for the majority of genes proportions studied (p<2.2 e^-16^).

**Table-2:** Virulence profile of avian pathogenic *Escherichia coli* isolates.

Virulence profile	Virulence factor number	Frequency n=43	Percentage	p-value
*iroN omp*T *iss hly*F *iut*A *sit*A	6	5	11.60	9.425×10^−16^***
*iro*N o*mp*T *hly*F *iss iut*A	5	1	2.30	2.2×10^−16^***
*iro*N o*mp*T *hly*F *iss sit*A	5	10	23.25	1.286×10^−12^***
o*mp*T *hly*F *iss*	3	1	2.30	2.2×10^−16^***
o*mp*T *hly*F *frz_orf4_*	3	6	14.00	4.482×10^−15^***
*pap*C *fel*A	2	1	2.30	2.2×10^−16^***
*sit*A	1	1	2.30	2.2×10^−16^***
None factor	0	18	41.80	1.203×10^−8^***

*** Very highly significant value

### Antibiotic resistance

Susceptibility study to 22 antibiotics belonging to seven chemical families of 36 isolates, from the collection of *E. coli* outcome colibacillosis lesions, revealed the sensitivity of a single isolate to all antibiotics. One isolate (2.7%) was resistant to a single molecule of antibiotic and 34 isolates (70.12%) to more than two antibiotics. In addition, a multidrug resistance observed on 53.5% of isolates covered more than 3 classes of antibiotics. In contrast, no resistance to colistin and imipenem was observed in studied isolates. The sensitivity and resistance profiles of the isolates tested are summarized in Table-3.

**Table-3:** Susceptibility and resistance rates to antibiotics of avian pathogenic *Escherichia coli* isolates.

Antibiotic	Number of isolates	Frequency and percentage of susceptibility (%)	Frequency and percentage of intermediate (%)	Frequency and percentage of resistance (%)	p-value
AMX	36	5 (13.8)	1 (2.8)	30 (83.3)	3.901×10^−14^***
AMP	36	5 (13.8)	1 (2.8)	30 (83.3)	3.901×10^−14^***
AMC	36	10 (27.7)	20 (55.6)	6 (16.7)	0.001503***
TIC	36	6 (16.7)	1 (2.8)	29 (80.5)	5.23110^−13^***
IPM	36	38 (100)	0	0	<2.2×10^−16^***
ATM	35	20 (57.1)	2 (5.8)	13 (37.1)	527×10^−5^***
CZ	34	9 (26.5)	5 (14.7)	20 (58.8)	0.0003404***
FOX	35	27 (77.1)	7 (20)	1 (2.9)	4.481×10^−11^***
CTX	36	21 (58.3)	2 (5.6)	13 (36.1)	1.148×10^−5^***
CAZ	30	15 (50)	2 (6.7)	13 (43.3)	0.0006426***
CFM	35	20 (57.1)	1 (2.9)	14 (40)	5.403×10^−6^***
CPO	19	16 (84.2)	1 (5.3)	2 (10.5)	5.829×10^−8^***
K	35	18 (51.4)	2 (5.8)	15 (42.8)	9.142×10^−5^***
GEN	36	34 (94.4)	1 (2.8)	1 (2.8)	<2.2×10^−16^***
TE	35	15 (42,9)	0	20 (57.1)	8.931×10^−7^***
CL	35	35 (100)	0	0	<2.2×10^−16^***
SXT	36	11 (30.6)	1 (2.8)	24 (66.6)	6.024×10^−8^***
NA	36	5 (13.8)	1 (2.8)	30 (83.4)	3.901×10^−14^***
PA	36	4 (11.2)	5 (13.8)	27 (75)	6.692×10^−10^***
CIP	35	14 (40)	0	21 (60)	4.129×10^−7^***
OFX	36	12 (33.4)	1 (2.8)	23 (63.8)	2.7×10^−7^***
PF	35	15 (42.9)	1 (2.8)	19 (54.3)	1.028×10^−5^***

The proportion comparison χ^2^ test performed for a value of α=5%;

*** Very highly significant value. AMX=Amoxicillin, AMP=Ampicillin, AMC=Amoxicillin+clavulanic acid, TIC=Ticarcillin, IPM=Imipenem, ATM=Aztreonam, CZ=Céfazolin, FOX=Cefoxitin, CTX=Cefotaxime, CAZ=Ceftazidime, CFM=Cefixime, CPO=Cefpirome, K=Kanamycin, GEN=Gentamicin, TE=Tetracycline, CL=Colistin, SXT=Triméthoprim+sulfamethoxazole, NA=Nalidixic acid, PA=Pipemidic acid, CIP=Ciprofloxacin, OFX=Ofloxacin, PF=Pefloxacin

Regarding beta-lactams, the results showed that the highest resistance rates were observed for aminopenicillins represented by ampicillin and amoxicillin and ticarcillin in the carboxypenicillin group with 83.3% and 80.5%, respectively. Average levels were noted with respect to other beta-lactams such as aztreonam (37.1%) and cefazolin (58.8%), cefotaxime (36.1%), cefixime (40%), and ceftazidime (43.3%), respectively, of the group of monobactams and cephalosporins of the first generation and third generation. The lowest resistance rates were recorded in the association amoxicillin-clavulanic acid (16.7%), the fourth-generation cephalosporins (cefpirome 10.5%) with the extreme value (2.9%) recorded for cefoxitin, the second-generation cephalosporin.

It should be noted that resistance to beta-lactams is associated with resistance to all other classes with the predominance of aminoglycoside with 14 isolates (38.8%) and beta-lactams-aminoglycoside-tetracycline-quinolones combination with 13 isolates (36.1%).

The analysis of the beta-lactam resistance profiles for all isolates according to CA-SFM [14] and Livermore *et al*. [18] showed that the main mechanism of resistance was the production of beta-lactamases. The majority of isolates (44.4%) carried broad-spectrum β-lactamases followed by the presence of ESBL on 36.1% of isolates and a single AmpC high-level cephalosporinase isolate.

Twenty-five strains (56.80%) carried at least one virulence factor and the profiles observed on the latter showed that the isolates were resistant to at least four antibiotics and also up to 16 antibiotics simultaneously.

The distribution of the isolates according to the presence of the virulence factors and the beta-lactam resistance profile (Table-4) showed that 56.30% of the broad-spectrum beta-lactamases profile was associated with the presence of five virulence genes *iro*N, *omp*T, *iss, hly*F, and *sit*A and that the majority of ESBLs (46.2%) carried the *omp*T and *hly*F genes and frz_orf4_ fragment.

**Table-4:** Virulence profile associated with beta-lactam resistance mechanism.

Mechanism virulence profile	BLSE (n=13)	Broad-spectrum beta-lactamase (n=16)	Cephalosporinase (n=1)
*iro*N *omp*T *iss hly*F *iut*A *sit*A	1 (7.7%) *p*1.509×10^−5^***	2 (12.4%) *p*3.612×10^−6^***	1 (100%)
*iro*N *omp*T *iss hly*F *sit*A	1 (7.7%) *p*1.509×10^−5^***	9 (56.3%) *p*0.0103**	-
*omp*T *hly*F *frz_orf4_*	6 (46.2%) *p*0.007982***	-	-
*pap*C *fel*A	-	1 (6.25%) *p*7.07×10^−7^***	-
*sit*A	-	1 (6.25%) *p*7.07×10^−7^***	-
None factor	5 (38.4%) *p*0.002935**	3 (18.8%) *p*1.566×10^−5^***	-

*** Very highly significant value;

** highly significant value

**Figure-2:**
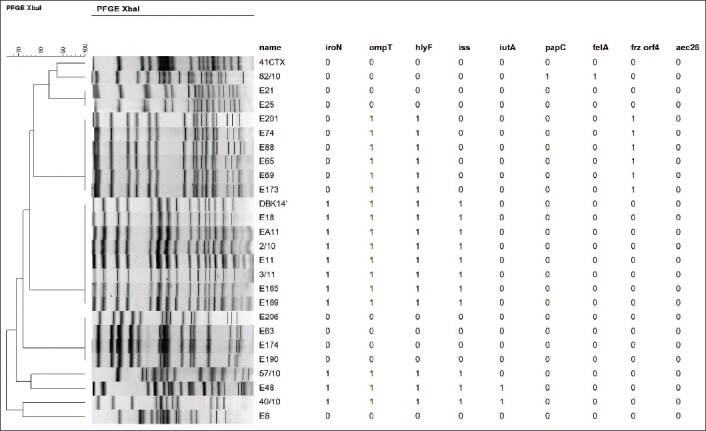
Molecular characterization and pulsed-field gel electrophoresis analysis of *Escherichia coli* isolates.

**Figure-3:**
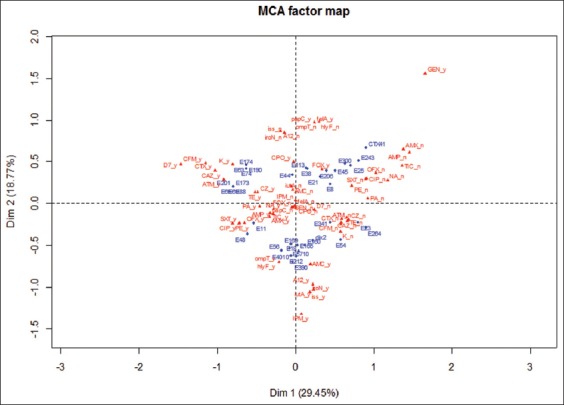
Graphical representation of the multiple correspondence analysis performed by the R software. Blue: Isolates; red: Virulence factors and antibiotics. The statistical data analysis by MCA has globally established a relationship between antibiotics resistance and virulence factors present in tested strains. The results of the ACM showed the first and second plans, respectively, expressing 29.45% and 18.77% of the total variability. The information contained on these plans is considered sufficient with 52.4% inertia value.

The analysis of genetic profiles by PFGE showed the presence of five different genetic profiles, which reveals a diversity of the 43 avian *E. coli* analyzed (Figure-2). MCA analysis has shown that virulence determinants seem to have a phenotypical relationship with antibiotics resistance such revealed by MCA analysis (Figure-3).

## Discussion

Our results showed that *E. coli* strains studied are diverse with different pathogenicity patterns and some of them are considered as potentially highly virulent since they harbored four or five virulence genes [10,19]. However, certain strains isolated of lesions may not be pathogenic since none virulence factor has been detected with Johnson *et al*. [10] and Schouler *et al*. [11] APEC criteria.

APEC strains can be genetically very diverse and have a distinct repertoire of virulence genes [4]. It appears from the literature that virulence factors are not all present in the same isolate and there is not a single or a set of specific virulence genes systematically associated with APECs, hence, the difficulty diagnosis and implementation of treatment targeting all isolates [10,20]. Moreover, many authors report genetically diverse populations of *E. coli* in field cases of colibacillosis [21-24] and overall in APEC [25]. Furthermore, according to Collingwood *et al*. [26], colibacillosis in birds can result from infection with isolates of a pathotype other than APEC. Besides, most of the disease associated with *E. coli* in domestic poultry is as much a consequence of increased host susceptibility due to stress, immune suppression, coinfection, or poor welfare. This leads to more “opportunistic” infections rather than the result of infection with a specific pathotype.

In Algeria, the prevalence of virulence-associated factors still poorly is known. Among these rare studies, Lounis *et al*. [27] have reported, in opposition to our results, that the most prevalent genes in APEC were *iut*A (90.6%) followed by *omp*T (86.9%) and *iss* (85.8%). While Laarem *et al*. [28] detected the presence of 4 isolates (13.8%) of avian *E. col*i carried one of Shig*a* toxin *E. coli*-associated genes *stx*1, *stx*2, and *ehxA* alleles. Likewise, in a recent study in Algeria [29], a set of plasmidic virulence genes (*iut*A, *fyuA*, *irp2*, *iro*N, *fimH*, *cvaC*, *traT*, *iss*, *sit*A, *omp*T, *hly*F, *cvaA*, *etsA*, *etsB*, *eitA*, and *tsh*) and chromosomal virulence genes (*sit*A *fyuA*, *vat*, and *ibeA*) associated with APEC have been detected, paradoxically, in fecal *E. coli* strains isolated from clinically healthy chickens.

An Egyptian studies have shown that among 91 non-repetitive *E. coli* isolates, 73 (80.2%) carried three or more of the APEC virulence genes *iro*N, *omp*T, *iss*, *iut*A, and *hly*F [30] and according to Mohamed *et al*. [31], *iss* gene was found in 72.2% of the examined extraintestinal pathogenic *E. coli* (ExPEC) strains from diseased broiler chickens. More than 90% of the total APEC examined possessed *iro*N, *omp*T, *hly*F, *iss*, and *iut*A, and 53.5% harbored plasmid pathogenicity islands. In Iran, eight different combination patterns of the virulence genes were detected among colibacillosis isolates [32]. In Zabol, as a border region of this country, 86.9% of isolates collected from chickens with colibacillosis were positive for *iss* gene [33]. For Paixão *et al*. [34], the iron uptake-related genes and the serum survival gene were more prevalent among APEC.

Despite the diversity of virulence gene profiles (*iro*N, *omp*T, *hly*F, *iss*, and *iut*A and others) observed in suspected isolates of colibacillosis in South Africa [35], this study revealed, in agreement with our findings, that the *iut*A gene was not systematically present in the various samples.

It should be noted that *pap*C, *fel*A (2.3%), and *aec*26 genes are rarely found or inexistent in our strains set. Cunha *et al*. [36] also yielded a low prevalence of some genes that are frequently described in APEC, such as *iss* (37%), *om*pT, and *hl*yF (8% each). Consequently, the occurrence and frequency of these markers may vary according to the geographic origin and year of the isolation.

Moreover, this study does not exclude that the strains without virulence factor could have both variants of these genes [37] or an arsenal of virulence factors not detected which would be responsible for colibacillosis lesions. Further investigation should be undertaken for confirmation. It should be noted that these non APEC strain are resistant to at least one antibiotic and several are multidrug-resistant. Indeed, according to Moreno *et al*. [38] and Maciel *et al*. [39], commensal *E. coli* can also generate extra intestinal lesions influenced by antimicrobial resistance. On the other hand, these APEC characterization tests seem limited according to Dziva *et al*. [40] since far too often, avian isolates are considered as APEC according to exclusively on their PCR-detected genotypic profile. However, this is a misleading strategy since avian isolates should only be characterized as APEC if their virulence has been confirmed in animal models validated for avian colibacillosis [4].

Antimicrobial susceptibility tests were performed to characterize phenotypic features of isolates. Through the years, increased use of antibiotics has been observed in diverse activities in our country, including growth promote (although regulated since 2006), preventive, or treatment care. This extensive and uncontrolled use of antibiotics, combined with easiness of access, mostly for veterinary practice, led to the development of antibiotic resistance.

A high percentage of multidrug-resistant *E. coli* was detected in this study. It is known that *E. coli* strains isolated from poultry frequently show multi-resistance to more than one antimicrobial drug [41] which represents a global concern.

In Algeria, antibiotic resistance has been further studied than virulence contents of APEC. The most recent reports have shown that *E. coli* isolates in poultry products harbor high levels of resistance to tetracycline and sulfamethoxazole (96.6%), ciprofloxacin (72%), and amoxicillin (65.5%) [28]. Halfaoui *et al*. [42] have isolated pathogenic *E. coli* strains from broiler chicken with colibacillosis in the central of Algeria that presented a high level of resistance to tetracycline (94.12%), flumequine (91.5%), sulfamethoxazole-trimethoprim (88.89%), enrofloxacin (86.27%), nalidixic acid (85.62%), ampicillin (83.01%), and doxycycline (75.81%).

Another result from APEC [28] also showed that the highest rates of resistance were against tetracycline (97.4%). High levels of resistance were again observed in the same study for sulfisoxazole (94.9%), trimethoprim-sulfamethoxazole (92.3%), ampicillin (89.7%), and ofloxacin (84.6%). Colistin (2.8%) and gentamicin (35.9%) seemed to be among the most efficient antibiotics against APEC isolates. The moderate resistance of gentamicin may be due to its illicit use knowing that this antibiotic is prohibited in veterinary medicine in Algeria. While, it was observed an emergence of mcr-1-mediated colistin resistance in *E. coli* isolates from poultry in Algeria [43]. Curiously, none ESBL was revealed in these studies while these enzymes, especially CTX-M-1 type, were previously detected since 2015 [44].

Similar to our findings in Morocco [45] and Central Ethiopia [46], extremely high levels of resistance to amoxicillin (90.9% and 100%, respectively) and trimethoprim + sulfamethoxazole (82.2%) were recorded and low frequencies of resistances were noted for gentamicin (24.8%) and colistin (2%) [45], similarly, at Italian findings of Sgariglia *et al*. [25]. Only these past 2 years, several other publications across the globe have signalized rising levels of antibiotic resistance in APEC isolated from colibacillosis such as Nepal [47] and Senegal [48].

Our results have shown that virulence determinants seem to have a relationship with antibiotics resistance at least in its phenotypic aspect. According to Da Silva and Mendonça [49], the topic on the link on resistance/virulence is complex, considering the diversity of antimicrobial resistance genes, virulence factors, bacterial species, and hosts. Most reports on his topic correlate the epidemiology of specific resistance genes with virulence genetic traits. This is a first step toward understanding whether there is a connection between resistance and virulence [6]. It is conceivable that virulence genetic determinants, if located on the same genetic platform as antimicrobial resistance genes (plasmids, transposons, and integrons), may be comobilized under antimicrobial selective pressure [49] and that both clones and plasmid may be involved on the dissemination of multiresistant *E. coli* [48]. Therefore, although antibiotic resistance is not in itself a virulence factor, in certain situations, it is a key factor in the development of infection, and it may be considered a virulence like factor in specific ecological niches which antibiotic-resistant bacteria are able to colonize [50].

## Conclusion

Our current study characterized the genetic contents of virulence and antimicrobial resistance in the *E. coli* strains isolated from broilers colibacillosis lesions in central of Algeria. Indeed, concerning APEC virulence characterization, this study remains a contribution and not definitive data for many other Algerian regions and does not represent the virulence gene content in the whole country.

However, the high frequency of antimicrobial resistance, associated with several virulence factors, in particular, the presence of ESBLs strains harboring until six virulence factors in these APEC strains, represents a potential public health problem that requires to maintain constant vigilance guidelines and regulations.

## Authors’ Contributions

NM designed and performed the study and wrote the manuscript. NC, AT, CAA, and SB managed the analyses of the study and were involved in data analysis. CS direct and supervised the project. All authors read and approved the final manuscript.
